# Effect of selenium supplementation on CD4 T-cell recovery, viral suppression, morbidity and quality of life of HIV-infected patients in Rwanda: study protocol for a randomized controlled trial

**DOI:** 10.1186/1745-6215-12-192

**Published:** 2011-08-13

**Authors:** Julius Kamwesiga, Vincent Mutabazi, Josephine Kayumba, Jean-Claude K Tayari, Richard Smyth, Heather Fay, Alice Umurerwa, Marcel Baziruwiha, Christian Ntizimira, Antoinette Murebwayire, Jean Pierre Haguma, Julienne Nyiransabimana, Donatille Habarurema, Veneranda Mukarukundo, Jean Bosco Nzabandora, Pascal Nzamwita, Ernestine Mukazayire, Edward J Mills, Dugald Seely, Douglas J McCready, Don Warren

**Affiliations:** 1 Rwanda Selenium Supplementation Clinical Trial Team, Kigali, Rwanda; 2 Interdisciplinary School of Health Sciences, Faculty of Health Sciences, University of Ottawa, Ottawa, Ontario, Canada; 3 Canadian College of Naturopathic Medicine, North York, Ontario, Canada; 4 Department of Economics, School of Business and Economics, Wilfrid Laurier University, Waterloo, Ontario, Canada

## Abstract

**Background:**

Low levels of serum selenium are associated with increased risk of mortality among HIV+ patients in East Africa. We aim to assess the effect of selenium supplementation on CD4 cell count, HIV viral load, opportunistic infections, and quality of life in HIV-infected patients in Rwanda.

**Methods and Design:**

A 24-month, multi-centre, patient and provider-blinded, randomized, placebo-controlled clinical trial involving 300 pre-antiretroviral therapy (ART) HIV-infected patients will be carried out at two sites in Rwanda. Patients ≥ 21 years of age with documented HIV infection, CD4 cell count of 400-650 cells/mm^3^, and not yet on ART will be recruited. Patients will be randomized at each study site using a randomized block design to receive either the selenium micronutrient supplement or an identically appearing placebo taken once daily. The primary outcome is a composite of time from baseline to reduction of CD4 T lymphocyte count below 350 cells/mm^3^ (confirmed by two measures at least one week apart), or start of ART, or the emergence of a documented CDC-defined AIDS-defining illness. An intention-to-treat analysis will be conducted using stepwise regression and structural equation modeling.

**Discussion:**

Micronutrient interventions that aim to improve CD4 cell count, decrease opportunistic infections, decrease HIV viral load, and ultimately delay initiation of more costly ART may be beneficial, particularly in resource-constrained settings, such as sub-Saharan Africa. Additional trials are needed to determine if micro-supplementation can delay the need for more costly ART among HIV-infected patients. If shown to be effective, selenium supplementation may be of public health importance to HIV-infected populations, particularly in sub-Saharan Africa and other resource-constrained settings.

**Trial Registration:**

NCT01327755

## Background

In sub-Saharan Africa, more than 30 million people are living with HIV/AIDS, malnutrition and food insecurity are endemic [1] HIV infection compromises the nutritional status of infected individuals and poor nutritional status can enhance progression of the disease [2]. The relationship between immune function and nutritional supplementation has been well described [3-6]. Studies have reported a high prevalence of nutrient deficiencies early in the course of HIV infection [7-9].

It is well understood that micronutrient deficiencies and HIV disease progression aggravate each other [10,11]. Among HIV-infected persons not receiving antiretroviral therapy (ART), observational studies have shown that low or deficient serum concentrations of several micronutrients are associated with low CD4 cell count, advanced HIV related diseases, faster disease progression, or HIV-related mortality [12-26]. Selenium is one essential nutrient necessary for endogenous antioxidant defense. Selenium deficiency, as indicated by low plasma selenium concentrations, is common among HIV-infected individuals [27,28].

While many observational studies on selenium in HIV-infected patients have been conducted in developed countries over the past decade, few have been conducted in sub-Saharan Africa. Clinical trial data on selenium supplementation is also limited in both developed and developing countries. There are two recent high-impact randomized clinical trials (RCTs) that have been conducted to assess the individual association of selenium supplementation on HIV viral load and CD4 cell count. The first trial, conducted in Miami by Hurwitz et al [29], found that selenium supplementation of 200 μg daily significantly suppressed the progression of HIV viral load and improved CD4 cell count after 9 months of treatment. The second trial, conducted in Tanzania by Kupka et al [30], found that selenium supplementation of 200 μg daily provided to HIV-infected pregnant women before and after pregnancy (between 12 and 27 weeks of gestation and 6 months after birth) had no significant effect on HIV viral load or CD4 cell count, but did significantly lower risk of infant death.

Although the results of these two clinical trials appear to contradict, it is important to recognize the inherent differences in their design, setting, and populations under study. Additional evidence from other settings and populations is still required to more accurately determine the effect of selenium supplementation on HIV viral load and CD4 cell count in HIV-infected individuals. Therefore, we have designed a randomized trial to examine the effect of selenium supplementation on CD4 cell counts in HIV-infected patients who are not yet on ART. This trial will take place in Rwanda, where an estimated 250,000 adults and children are living with HIV [31], of which only 50,000 are receiving ART [32].

## Methods and Design

### Funding

Global Benefit has sponsored this trial. The trial sponsors and investigators will make financial assurance statements to the concerned bodies (Ministry of Health's National Ethics Commission, National Research Commission) on the availability of funds for the completion of the trial.

### Registration

This trial has been registered with ClinicalTrials.gov. The registration number is NCT01327755.

### Study Design

**Figure 1:**
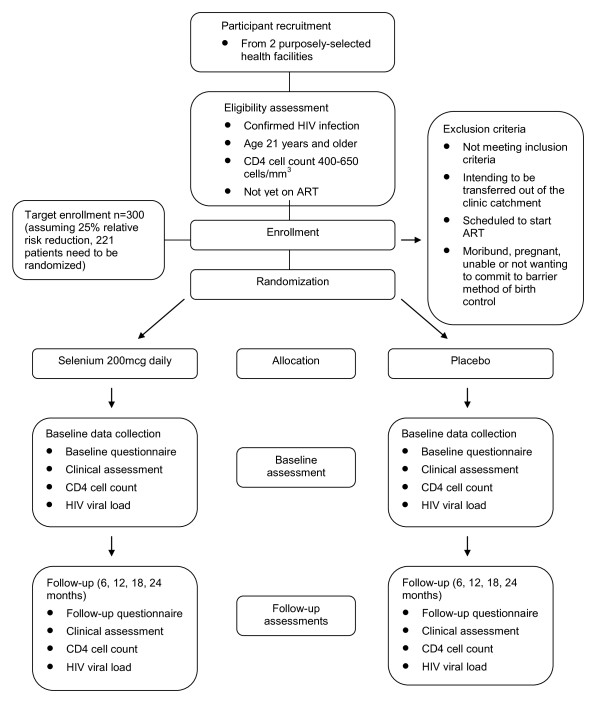
**Study flow diagram**. This figure displays the intended recruitment and measurement points in this trial.

This study is a 24-month, multi-centre, patient, provider and analyst-blinded, randomized, placebo-controlled clinical trial involving 300 pre-ART HIV-infected patients in Rwanda. (See Figure 1 for a study flow diagram.)

### Objectives and Hypothesis

The primary outcome of the study is time from baseline to reduction of CD4 T lymphocyte count below 350 cells/mm^3^ (confirmed by two measures at least one week apart), or start of ART, or the emergence of a documented CDC-defined AIDS-defining illness. We hypothesize that selenium supplementation in pre-ART patients will improve CD4 cell counts, decrease opportunistic infections, decrease HIV viral load, and delay ART initiation.

### Setting and Participants

Patients will be recruited at two purposely-selected health facilities that offer care and treatment for HIV/AIDS patients in Rwanda. These facilities have been chosen due to the feasibility of recruiting all patients within a 3-4 month period and the feasibility of coordination.

Patient eligibility will be restricted to HIV-infected adults 21 years of age and older at study enrolment. Only patients not yet eligible for ART will be included. Only patients with CD4 cell count between 400 and 650 cells/mm^3 ^will be selected because they are at similar immunological level and hence they will not be eligible to start ART treatment at the start of the study. Eligible participants must also be willing to practice a barrier method of birth control at all times. Written informed consent will be required from patients to participate in this study.

Eligible patients will be identified from patient registers. These patients will be informed about the study during a regular scheduled clinic visit or through home visits by site staff not otherwise involved in data collection. Patients who are interested in the study will be provided with further details and consent procedures. Those fitting the inclusion criteria will be enrolled and followed for 2 years. Study assessments will occur at baseline, 6, 12, 18, and 24 months.

Patients will be excluded if they are intending to be transferred out of the clinic catchment area before study ends and/or if they are scheduled to start ART. Moribund patients, pregnant women, and those unable or not wanting to commit to barrier method of birth control will also be excluded.

### Randomization

Participants will be randomized using a simple randomized block design to receive either selenium or an identically appearing placebo to be taken once daily for 24 months. The Rwandan Ministry of Health recommends the use of co-trimoxazole, a sulfonamide antibiotic combination of trimethoprium and sulfamethoxazole used for the treatment of a variety of bacterial infections, for all HIV-infected patients. Therefore, all participants will also receive co-trimoxazole irrespective of experimental assignment. Participants who do not return to the clinic as scheduled will be followed up at home.

Study participants will be identified by a unique study identification number. The randomization schedule will be prepared by the product manufacturer, Seroyal, and the randomization sequence will be concealed from the study investigators and providers. Study participants will be assigned a specific allocation number. An unblinding list will be kept at the site of the manufacturer and will be provided as needed to the Data and Safety Monitoring Officer (DS). Site study personnel (investigator and clinical personnel monitoring the safety and laboratory assay results) and study participants will be blinded with respect to the allocation. Unblinding of an individual study participant will be indicated in the event of a medical emergency where the clinical management/medical treatment of the study participant could be altered by knowledge of the group assignment allocation of the investigational product.

### Intervention

The trial intervention will consist of capsules containing 200 mcg of selenium in the form of selenium yeast, which contains selenomethionine. Capsules will be provided in bottles of 30 (one months supply) and participants will be instructed to take one capsule daily. Selenium will be stored in a dry cool location during the course of the trial. Shelf life of the product is well beyond the two-year time period expected for the trial duration. To ensure optimal adherence, participants will receive adherence counseling at baseline and when picking up refills on a monthly basis. Additional adherence counseling will be provided to patients who have sub-optimal adherence.

Selenium capsules are supplied by Seroyal, a Canadian Nutraceutical company. The sponsors of the trial and the investigators will be responsible for ensuring that the investigational pharmaceutical product is safe. Comparator products (placebo) supplied for the clinical trial will be of proven quality and will be available to be verified by the National Bureau of Standards. Records will be kept of information about the shipment, delivery, receipt, storage, return, and destruction of any remaining pharmaceutical products. The investigators will not supply the investigational product to any person not targeted to receive it.

### Outcomes

The primary outcome of the study is a composite outcome involving reduction of CD4 T lymphocyte count to below 350 cells/mm^3^ (confirmed by two measures at least one week apart), or start of ART, or the emergence of a documented CDC-defined AIDS-defining illness. Secondary outcomes includes viral suppression at 6, 12, 18, and 24 months; quality of life; weight gain; presence of opportunistic infections and mortality.

### Measurement of Outcomes and Other Variables of Interest

#### Patient Interviews

Trained nurses will use a structured questionnaire to collect data on patients' demographics at baseline. Additionally, at baseline and at each follow-up visit, a questionnaire will be used to collect information on psychosocial factors, access to care and treatment, attitudes towards and experiences with the supplementation, quality of life, self-efficacy, nutrition, opportunistic infections, and adherence to the study protocol. The questionnaire will be available in English and Kinyarwanda. Nurses will also use a data abstraction tool at baseline and at each follow-up visit to obtain information on height, weight, blood pressure and other clinically relevant information.

#### HIV Viral Load and CD4 Cell Count

At baseline, and at month 6, 12, 18 and 24, blood draws will be done for assessment of CD4 cell count and HIV viral load. Blood samples will be collected into EDTA tubes (Becton Dickinson, San Jose, CA, USA). The viral load samples will be transported and analyzed at the National Reference Laboratory within 4 hours; plasma will be separated from cells by centrifugation at 200 g, aliquoted and stored at -70°C until the real time PCR will be performed using Cobas TaqMan 48 (Roche Diagnostic Systems, NJ, USA) that has a detection limit of 40 RNA copies/ml. The RNA will be extracted from the plasma with chloroform, followed by alcohol precipitation and dilution with the HIV Monitor specimen diluent (100 μl). Amplification and detection of the extracted RNA (40 μl) will be performed in accordance with the manufacturer's instructions. CD4 cell count will be measured at the National Reference Lab, or the site laboratory, using FACS Count (Becton Dickinson immunocytometry).

### Sample Size Calculation

We chose our CD4 depletion event rate based on the work of the CASCADE cohort which displays an average CD4 depletion of 114 (32-229) cells/μl per year and where 54% of individuals had a decline > 100 cells/μl per year if CD4 evaluations were rare, as in Rwanda [33]. We expect that the majority of participants in our study, about 60%, will enter the study with a CD4 at risk of reaching 350 cells/μl within the first year as most evidence demonstrates that patients in East Africa initiate treatment due to symptomatic HIV with a median CD4 of 141 to 169 (depending on source). Thus, patients with a high CD4 will be somewhat more rare to enroll [34,35].

We applied several sample sizes to display that even small changes in the relative risk of the intervention will yield a large impact on the required sample size (see Table 1). Our three sample size assumptions are based on the likelihood of an intervention delivering a small, moderate or large effect (relative risk reduction of 10, 25, and 50%). Given that we do not have strong evidence from previously completed trials, we are assuming the event of depleting CD4 status will occur in 60% of control patients at one year and 60% of experimental patients at one year if the intervention delivers no effect. Table 1 provides the estimates required for power of 80% and an alpha of 0.05.

**Table 1:** Sample Size Calculation estimates

RRR	Control event rate	Experimental event rate	Sample size	Power
10%	60%	55%	2312	80%
25%	60%	44%	314	80%
50%	60%	29%	76	80%

We employed several methods to make these calculations. The below estimates are based on Markov Chain Monte Carlo Methods whereby we explored the impact of adherence/retention to the intervention and examined the differing possible risks of an underlying disease event. We performed 5,000 simulations for each estimate. As we employ informed estimates, using a Bayesian profile, our estimates may differ slightly compared with other software approaches. However, they should not differ to an important amount.

Based on these calculations and our resources, we aim to enroll 300 patients in the trial. This leads to an RRR slightly lower than 25% and represents a very important reduction in events. Recognizing that no single trial can provide definitive evidence of effectiveness, this trial will contribute to the overall evidence of selenium supplementation for CD4 maintenance [36,37]. If control patients demonstrate lower rates of compliance to the intervention due to a lack of effects of, say, 20%, the power is increased to 96% and an alfa of 0.25. At the conclusion of our trial, we will conduct a meta-analysis to determine the overall power that current evidence contributes to answering this clinical question.

### Analysis Plan

Analysis will be conducted jointly by the study team in Rwanda and in Canada using standard statistical software. The analysis and reporting of the results with follow the CONSORT guidelines [38]. The statistician/data analyst will be blinded to the study group. The process of patient selection and flow throughout the study will be summarized using a flow-diagram (See Figure 1). The analysis results of patient demographics and baseline outcome variables (both primary and secondary) will be summarized using descriptive summary measures: expressed as mean (standard deviation) or median (minimum-maximum) for continuous variables and number (percent) for categorical variables. We will adopt an intention-to-treat principle to analyze all outcomes, meaning that data from participants will be analyzed according to the group to which they were randomized even if they do not receive the allocated intervention. We will also use multiple-imputation to handle missing data. We will use the T-test for comparing groups on continuous outcomes and the chi-squared test for binary outcomes. We will consider a threshold of 0.05 as statistical significance. For all group comparisons, the results will be expressed as effect (risk ratio for binary outcomes), corresponding two-sided 95% confidence intervals and associated p-values. P-values will be reported to three decimal places with values less than 0.001 reported as < 0.001. Because the primary outcome is a composite outcome, we will assess heterogeneity between the included outcomes [39,40]. Further, adjusted analyses using the following baseline covariates (age, gender, nutritional status) will be performed using regression techniques to investigate the residual impact of key baseline characteristics on the outcomes. Goodness-of-fit will be assessed by examining the residuals for model assumptions and chi-squared test of goodness-of-fit. All analyses will be performed by a professional statistician.

### Adverse Events

This study will use the standard level of expedited adverse event (AE) reporting as defined in the Division of AIDS (DAIDS) AE Manual. At this level, this study will report all AEs following any exposure to the investigational product. AE follow-up will be reported on a standardized form during the protocol-defined AE reporting period, which will be the entire study duration for an individual participant (from study enrollment until study completion or study discontinuation of a participant for any reason). After the end of the protocol-defined AE reporting period, sites will report serious, unexpected, clinical suspected adverse drug reactions or if the study site staff becomes aware of the event on a passive basis (e.g. from publicly available information).

AEs will be managed in accordance with good medical practices by the clinical study team who will assess and treat the study participant as appropriate, including referral. All study participants experiencing AEs, regardless of severity, will be followed until satisfactory resolution, return to baseline, or until the toxicity is presumed to be irreversible. If at the end of the study, an AE (including clinically significant lab abnormality) which is considered possibly, probably or definitely related to the investigational product is unresolved, follow-up will continue until resolution if possible and the study participant will be referred. If treatment and medical care is required as a result of harm caused by the investigational product or study procedures, this will be provided free of charge to the participant.

### Ethical Considerations

This trial will be conducted in compliance with the protocol approved by the institutional review boards of the Canadian College of Naturopathic Medicine and Wilfred Laurier University in Canada, and the National Ethics Committee (NEC) in Rwanda. No deviation from the protocol will be implemented without the prior review and approval of the IRB except where it may be necessary to eliminate an immediate hazard to a participant. In such case, the deviation will be reported to the IRB as soon as possible.

A signed consent form will be obtained from each participant. The consent form describes the purpose of the trial, the procedures to be followed and the risks and benefits of participation. A copy of the consent form will be offered to the participant. Trial participants will receive 1,000 Rwandan Francs (equivalent to ~ $ 1.67 US dollars) per month for their travel to and from the clinic for interviews, lab work-up and picking up their supplement. In addition to this compensation, in the event that the study shows benefit, Global Benefit (the trial funder) is committed to supplying free supplementation to all participants in the trial for a period of at least one year following study completion.

### Quality Control and Quality Assurance

The sponsor and investigators will be responsible for implementing and maintaining quality assurance and quality control systems with written standard operating procedures to ensure that the trial is conducted and data are generated, documented, and reported in compliance with the protocol, good clinical practice, and the applicable regulatory requirements. The sponsor will also be responsible for securing agreement from all involved parties to ensure direct access to all trial related sites, source data and documents, and reports for the purpose of monitoring and auditing by the sponsor, and inspection by domestic and foreign regulatory authorities.

## Discussion

Micronutrient interventions that aim to improve CD4 cell count, decrease opportunistic infections, decrease HIV viral load, and ultimately delay initiation of more costly ART may be beneficial, particularly in resource-constrained settings, such as sub-Saharan Africa. It has been shown that selenium is deficient in HIV-infected populations [27,28]. For instance, a study conducted among HIV-infected children in Rwanda found that close to 40% had sub-optimal levels of selenium [41]. Data assessing the efficacy of selenium supplementation in randomized controlled trials is limited. Additional trials are needed to determine if selenium supplementation can delay the need for ART among HIV-infected patients. If shown to be effective, selenium supplementation may be of great public health importance to HIV-infected populations, particularly in sub-Saharan Africa and other resource-constrained settings.

## List of Abbreviations

AE: adverse event, AIDS: acquired immune deficiency syndrome, ART: antiretroviral therapy, CD4: cluster of differentiation 4, DAIDS: Division of AIDS, EAE: early adverse event, FDA: US Food and Drug Administration, HIV: human immunodeficiency virus, IRB: institutional review board, ITT: intention to treat, NEC: National Ethics Committee; NRL: National Reference Laboratory, PCR: polymerase chain reaction, RNA: ribonucleic acid, SEM: structural equation modeling.

## Competing interests

The authors declare that they have no competing interests.

## Authors' contributions

JK, VM, JK, JCK, RS, HF, AU, MB, CN, AM, JPH, JN, DH, VM, JBN, PN, EM, EJM, DS, DJM, DW conceived and designed the study. JK, VM, JK, JCK, RS, HF, AU, MB, CN, AM, JPH, JN, DH, VM, JBN, PN, EM, EJM, DS, DJM, DW interpreted the results of the literature search. JK, VM, JK, JCK, RS, HF, AU, MB, CN, AM, JPH, JN, DH, VM, JBN, PN, EM, EJM, DS, DJM, DW drafted the manuscript. All authors read and approved the final manuscript.
